# Tracking the push towards extinction: combining dispersal and management data to monitor Asian longhorned beetle eradication in the U.S.

**DOI:** 10.3389/finsc.2023.1286935

**Published:** 2023-12-07

**Authors:** Robert Talbot Trotter, Josie K. Ryan, Jennifer L. Chandler, Scott Pfister

**Affiliations:** ^1^ Northern Research Station, U.S. Forest Service, United States Department of Agriculture, Hamden, CT, United States; ^2^ Asian Longhorned Beetle Cooperative Eradication Program, Plant Protection and Quarantine, Animal and Plant Health Inspection Service, United States Department of Agriculture, Amityville, NY, United States; ^3^ Science and Technology, Plant Protection and Quarantine, Animal and Plant Health Inspection Service, United States Department of Agriculture, Buzzards Bay, MA, United States

**Keywords:** invasive species, dispersal kernel, risk management, eradication optimization, risk inference, forced extinction

## Abstract

**Introduction:**

Based on the threat posed by the Asian longhorned beetle (*Anoplophora glabripennis* Motschulsky), many countries including the United States have adopted policies of eradication. The eradication of infestations that cover hundreds of square kilometers can require multiple visual surveys of millions of individual trees. At these scales, eradication may take several decades and span multiple beetle generations. During this period the infestation of new trees adds spatially-explicit risk to the landscape while surveys and the removal of infested trees reduce it.

**Methods:**

To track dynamic risk on the landscape we have developed the Asian Longhorned Beetle Hazard Management and Monitoring Tool. The geospatial tool combines data documenting; the locations, levels of infestation, and dates of detection of infested trees; the locations, methods, and timing of survey and host removal activities; and a reconstruction of beetle movement within the infested landscape to generate annual spatial estimates of infestation risk based on the combination of beetle dispersal and survey and host removal activities.

**Results:**

The analyses of three eradication programs highlight similar patterns in risk through time with risk peaking at the time infestations are detected and declining as management activities slow beetle spread and reduce risk through surveys. However, the results also highlight differences in risk reduction among the eradication programs associated with differences in beetle dispersal among infestations and the size of the infested landscape, highlighting the importance of applying local information to structure eradication programs.

**Discussion:**

The Asian Longhorned Beetle Hazard Management and Monitoring Tool provides a quantitative repeatable approach to tracking changes in infestation risk using local beetle behavior and management efforts. In addition to this, the tool may provide a structure to optimize eradication efforts by allowing managers to estimate expected risk reduction based on proposed survey and host removal strategies.

## Introduction

The Asian longhorned beetle (*Anoplophora glabripennis*) could be considered an exemplar invasive species for the challenges it poses; it is easily transported in both live and cut wood (1, 2), has a broad host-range and climate tolerance (3), and has a high potential to cause economic and environmental harm (2, 4). The ability of the beetle to survive shipment in solid wood packing materials such as crates and pallets, and the use of infested material in international shipping has led to the introduction of the beetle into the U.S., Canada, and parts of Europe including Austria, Belgium, Finland, France, Germany, Italy, Montenegro, Turkey, the Netherlands, and the United Kingdom (5). *Anoplophora glabripennis* is known to feed on members of at least 15 *genera* (6) including Acer, Populus, Salix and other species that make up 30% of the standing forest in the eastern United States. An early study by Nowak et al. (7) found that the beetle poses a threat to 30% of the urban tree cover in the United States with the potential to cost municipalities an estimated $669 billion (7). Feeding by the beetle causes tree decline and a weakening of the physical structure of the tree which can lead to broken branches and trunks, posing a threat to property and safety. With the threat posed by the beetle to forested landscapes, the United States, Canada, and members of the European and Mediterranean Plant Protection Organization (EPPO) have adopted policies to prevent its introduction and to eradicate populations before they can become permanently established (8).

The process of biological invasion has often been described as a process with three phases; transport, establishment, and spread (9) and the strategies used to manage species invasions typically mirror these phases. In the case of the Asian longhorned beetle, phytosanitary methods such as International Standards for Phytosanitary Measures (ISPM) No. 15 (International Plant Protection Convention) set standards for the treatment of solid wood packing to prevent the movement of infested wood and ensure beetles in the wood do not survive to emerge and establish. However, once a population has been introduced to a landscape, management efforts must shift towards eradication to prevent the second phase; establishment.

Eradication efforts are most effective in this transition period between arrival and the establishment of a resilient population. At small population sizes a species may be more subject to extinction due to environmental, demographic and population stochasticity (10–13). The Allee Effect, the process by which small populations will tend towards extinction (14, 15) can also be leveraged to accelerate or facilitate eradication, and this approach has been applied to multiple invasive species including the Mediterranean fruit fly (16) and the suppression of satellite populations of species like the spongy moth (*Lymantria dispar*) by disrupting mate finding (17, 18).

In the case of the Asian longhorned beetle, methods to facilitate mating disruption have not been found, and the beetle’s tolerance of a wide range of environmental conditions (19, 20) works in favor of establishment. However, the rate of spread in infested landscapes has been relatively slow providing an opportunity to eradicate populations. In small infestations such as those found in Boston, Massachusetts and Paddock Wood in southern England (21), both of which included only a handful of infested trees in small, regulated areas, the complete removal of host has been a successful and efficient eradication method. In larger, more heavily–wooded landscapes, however, the challenge becomes more complex.

When infestations of the Asian longhorned beetle were found in central Massachusetts, Ohio, and South Carolina, the beetle populations had spread more broadly leading to the creation of regulated zones 285, 147, and ~150 square km in size (respectively). These regulated areas include urban, peri-urban, wooded, and agricultural regions that can include thousands of individual landowners and millions of host trees. While full host removal could be an effective eradication strategy, the size and complexity of the landscape may make the removal of all host trees in the regulated area neither economically feasible nor socially acceptable. As a result, eradication efforts in Ohio, Massachusetts, and South Carolina have focused on the use of surveys to find and remove individual infested trees with the goal of reducing population densities to push the population towards extinction. However, in these large landscapes a complete survey of the landscape may require multiple years, and as beetle populations are reduced finding the increasingly rare remaining infested trees and assessing the remaining risk become significant challenges.

To track the push of a species towards extinction there is a need to integrate information on the biology of the species that act to increase risk (reproduction, generation time, and dispersal), and the application of survey and host removal activities which reduce risk. The Asian longhorned beetle provides a unique opportunity to integrate these two processes by leveraging a previously-developed model of beetle dispersal (22–24) with data documenting eradication program surveys and tree detections collected by cooperative eradication programs in the United States. Bringing these data together may effectively link the biology and management of the beetle to quantitative changes in infestation risk on the landscape. This in turn can provide a structure to track progress toward eradication. The quantified connection between biology and management may also provide a way to evaluate future management efforts and optimize eradication resources.

Here we describe the basic structure and application of a tool to quantify and integrate the increase in risk resulting from beetle spread with the decrease in risk associated with management activities. The resulting tool is called the Asian Longhorned Beetle Hazard Management and Monitoring (ALBHMM) 2.0 and is available as a set of standalone programs and extensions for GIS. Using this system, we address two primary questions. First, are there differences in dispersal patterns and the distribution of risk among three different Asian longhorned beetle infestations in the United States, and second, can the integration of risk from dispersal and eradication via surveys and tree removals be used to track change in the intensity and distribution of risk on infested landscapes?

## Materials and methods

Background - The first documented detections of Asian longhorned beetles outside of Asia occurred in 1992 in both the United States and Canada when live beetles were found in crating and dunnage in warehouses. In 1996 a city resident in New York City found the first breeding population (25) and a program to eradicate the population was rapidly developed (more information on the history of this infestation can be found in 2). Since 1996 additional breeding populations have been found in North America and Europe including populations in the U.S. in New York outside of New York City, New Jersey, Ohio, Illinois, South Carolina, and Massachusetts.

In the United States efforts to eradicate infestations are managed by a locally operated Cooperative Asian Longhorned Beetle Eradication Program. These programs are managed cooperatively by the United States Department of Agriculture (USDA) Animal and Plant Health Inspection Service (APHIS), state departments of Forestry, Agriculture, and Environmental Protection, and local municipalities and public works departments. As of 2023, breeding populations of the Asian longhorned beetle have been successfully eradicated from Chicago Illinois, Boston Massachusetts, the state of New Jersey, two townships in Ohio (Stonelick and Monroe), and Brooklyn, Queens, Manhattan, Staten Island, and Islip in New York. Currently there are four active Cooperative Asian Longhorned Beetle Eradication Programs in the U.S. which are working to eliminate populations of the beetle from Ohio, Massachusetts, New York (Long Island), and South Carolina. The following analyses utilize data from the Ohio, Massachusetts, and South Carolina infestations. Data for New York is not readily available due to changes in data archiving methods and requirements over time. Work is underway to consolidate New York data to allow these analyses to be applied.

Surveys and Eradication Efforts in the U.S. – There is considerable variation among the four active U.S. programs in how survey activities are prioritized and executed due to differences in staffing, landscape structure, the age and progress in each of the programs, and a variety of other location-specific factors. Despite these differences, however, the programs share a common eradication methodology based on visual surveys of all host trees within the regulated area. Briefly summarized, individuals on survey crews will visually survey host trees (host list defined via Federal Register available at Federal Register:: Asian Longhorned Beetle: Update List of Regulated Articles) for signs of infestation (described in the following section). The time spent on a given tree is a function of the size and complexity of the tree and surveys on individual trees are conducted until the surveyor is satisfied that all suitable and visible portions of the tree have been assessed. Surveys are conducted year-round, and the rate of survey progression across the landscape can vary as a function the density and abundance of host trees, variation in physical access to land, and changes in staffing and disruptions to survey programs (such as those resulting from COVID-19). Survey effort is therefore a function of the total number of person-hours required to complete surveys for a given location. Survey quality is standardized both by the exchange of training and surveyors among eradication programs and with the use of validation surveys in which survey crews are tested on their ability to detect known and intentionally-placed signs of infestation in the field. In large infestations surveyors may be tasked with carrying out visual inspections of millions of individual trees and complete coverage of the landscape can take multiple years. Efforts to find more rapid and less-costly survey methods have included the evaluation of detection dogs (26, 27), acoustic surveys (28, 29), and baited traps (30–32). However, none of these methods has yet proven effective at a landscape-scale. Currently, visual surveys remain the foundation of eradication programs (33).

The Infestation Cycle and Visual Detection of Infested Trees– The infestation cycle begins when mated females chew pits in the bark of suitable host trees and insert a single egg into the cambium, leaving a distinctive scar on the bark (34). Eggs hatch within a few weeks and the first-instar larvae feed on cambial tissues. Feeding continues in the cambium through the first three instars after which the larvae move into the xylem (35). Feeding by larvae in the xylem leaves tunnels approximately 1 cm in diameter which weaken the structure of the tree directly by removing wood, and indirectly by facilitating decay. Once in the xylem, larvae complete at least 2 (though more are possible) additional instars to achieve a minimum weight range required for pupation (34). When temperatures are suitable (19) the larvae will chew horizontally through the xylem, stopping about 1 cm below the bark to chew a horizontal pupal chamber where the beetles pupate with their heads oriented towards the bark (34). When the adults emerge, they remain in the pupal chamber for 10-14 days to fully sclerotize (36) after which they chew out of the tree leaving a distinctive round exit hole.

The oviposition pits chewed by females in the bark of host trees, the exit holes produced by emerging adults, and the sawdust and frass pushed out of galleries by larvae are the visual cues used by survey crews to find and identify infested host trees. Survey crews may include personnel who survey trees from the ground using binoculars, and/or tree climbers who can more thoroughly access the tree canopy. In both cases, surveyors search the surface of the trunk and branches for signs of infestation (oviposition pits, exit holes, galleries), the efficacy of the survey is limited by the portion of the canopy that is visible. Other conditions including weather, lighting, season (leaf on or off), tree architecture, and the training and experience of the survey crews also play a role in determining survey efficacy. Past program assessments have indicated that ground surveys have an estimated detection efficacy of 30% while climbing surveys efficacy is estimated to be 70% based on the combination of the proportion of the surface that is visible and the ability to detect damage. With limited efficacy, multiple surveys of an area may be necessary.

As surveys are conducted, survey staff document the locations, methods (ground or aerial surveys), and dates of survey completion for a given location. Survey data is collected in a geographic information system and stored in a geodatabase as a collection of polygon features. When infested trees are found, their location, species, size (dbh), date of detection, and level of infestation is recorded as fields in a geodatabase point layer. Infestation levels are categorized and documented as A, B, C, or D such that A-level trees include oviposition pits but no exit holes, B trees have 1-10 exit holes, C trees have 11-100 exit holes, and D trees have more than 100 exit holes.

Model Structure – The term risk can be used in numerous ways. Here we define risk as the probability that a specified location (in this case a hectare) is infested, i.e., the hectare includes at least one infested tree. In the analyses described below, risk is estimated for each hectare in a 400 x 400-hectare (40 x 40 km) landscape. The use of this 160,000-hectare-scale provides a balance between the computational requirements of the analyses, the size of the region analyzed relative to the size of the landscape with measurable risk, and the scale at which landscapes are surveyed and managed by eradication programs in the United States. The computation of risk values for each hectare is carried out in four steps.

First, at each location, records of the locations and levels of infestation of each infested tree are used to reconstruct the spread of the beetle on the landscape. This inter-tree pattern of dispersal is used to estimate the two-dimensional dispersal kernel to quantify the estimated probability of dispersal risk around a given infested tree based on distance and direction using the process described in Trotter III and Hull-Sanders (22) and Trotter III et al. (23). Dispersal reconstructions and the estimation of the dispersal kernel are calculated using the tool ALBRisk 1.3, a modified version of ALBRisk 1.1 which is described in greater detail in Trotter III et al. (24). Briefly summarized, ALBRisk 1.3 is an analysis tool which uses the locations of known infested trees, the level of infestation for each tree, and explicit sets of rules to connect each infested tree on the landscape with another tree likely to have acted as the source for the infestation. The result is a graph of connections or adjacencies among infested trees representing a collection of dispersal events on the landscape. The tool is written in MatLab and is accessible as either a suite of scripts or as a standalone executable file. ALBRisk 1.3 differs from ALBRisk 1.1 with the addition of functions that save and output the data used in steps two and three below. The methods used to reconstruct dispersal and estimate the dispersal kernel remain consistent with those of ALBRisk 1.1.

In the second step, the year in which each tree on the landscape became infested is estimated and assigned to the tree. The year assigned is based on the year the tree was detected, and the level of infestation. In the case of the following analyses, A trees are assumed to have become infested the same year they were detected. B, C and D trees are assumed to have become infested 2, 4, and 8 years before detection. The first tree to have become infested (Origin tree used by ALBRisk) is assumed to have been infested 10 years before detection, an estimate that is consistent with tree ring analyses conducted by APHIS. The assumed lag between detection and infestation can be varied based on changes in generation time and estimated population growth rates, and while these factors merit additional study they are outside the scope of these analyses.

Third, a hectare-scale raster layer is generated for each year of the analysis starting with the first year a tree became infested and continuing through the current year. These annual layers are populated with the trees that became infested in the specified year, and the 2-dimensional dispersal kernel is applied to the location of each infested tree. While the shape and distribution of risk around each tree, based on the dispersal kernel are the same, the intensity of risk is mediated by the assumed number of dispersing females emigrating from each tree. As described in Trotter III et al. (23) the number of females assumed to emigrate is based on the level of infestation and the assumed dispersal rate. For these analyses the high dispersal rate was used such that A, B, C, and D trees produce 0.31, 3.1, 31, and 134 dispersing females (based on the maximum empirical dispersal estimates described in Trotter III et al. (23). Cumulative risk of dispersal is calculated for each hectare in a specified year based on all the trees infested within the given year, using the methods described in Trotter III et al. (24). The result is a set of annual layers that estimate the additional risk due to the infestation of new trees for each specified year. The risk value for each hectare at location (x,y) in year t is represented by a_(x,y,t)_ in the following equations.

**Figure 1:**
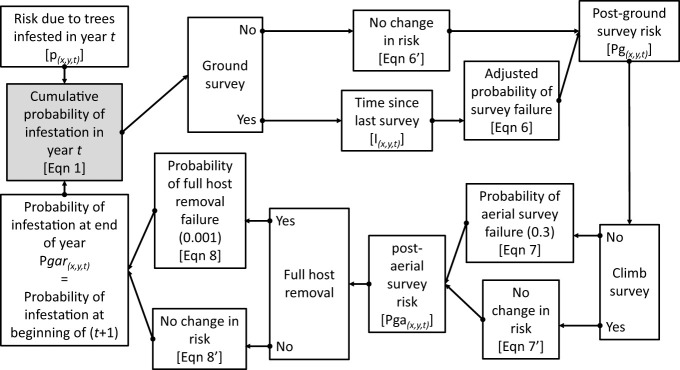
The Asian Longhorned Beetle Hazard Management and Monitoring (2.0) tool follows a computational loop as shown in the flow diagram. Variables and equations noted are described in greater detail in the Methods section. Note that the final estimate of risk at the end of the year is used to provide the input value to start calculations in the following year.


In the fourth step, the annual layers of infestation risk are compiled sequentially and cumulatively, such that the total accumulation of risk for each hectare is estimated for each year based on the locations and levels of infestation of all trees in the current and prior year, and risk is discounted by surveys and host removal activities in each year. This process is shown graphically in **Figure 1** and is described by the following equations. For clarity we use several conventions. The capital letter P denotes risk defined as the probability of infestation (or probability of beetle presence) and the letter A denotes the probability of beetle absence. Upper case risk parameters (P and A) represent the total cumulative risk for a location (x,y) in a specified year (t). Lower case letters (p and a) denote risk specific to a given year such that the cumulative probability of beetle absence in hectare (x,y) in year (t). Cumulative probabilities are calculated using the general form:

Equation 1 $$A_{\left(x,y,t\right)}=\;a_{\left(x,y,t\right)}*\;\left(1\;–\;Pgar_{\left(x,y,t-1\right)}\right)$$

Where P*gar*_(x,y,t-1)_ is the probability that the hectare at (x,y) was infested at the end of the prior year, and ax,y,t is the probability beetles are absent at location (x,y) based on infestations occurring in year t. Note that the value A_*t*_ represents the cumulative probability of infestation risk at the beginning of year t based on trees that became infested in year t, but prior to any management activity at (x,y) in year t. Using this function the probability that a given hectare (x,y) is infested at the end of year (t) is estimated by


Equation 2 $$P_{\left(x,y,t\right)}=\;1\;–\;A_{\left(x,y,t\right)}$$

Where P_(x,y,t)_ equals the cumulative probability that the hectare centered on (x,y) was infested at the end of year t. Risk can also be reduced for a specified location in a given year based on management efforts including surveys and host removals. The risk of infestation at the end of year t is estimated by combining the probability of infestation with the beetle elimination due to management activity and is described by the general form:


Equation 3 $$Pm_{\left(x,y,t\right)}=\;P_{\left(x,y,t\right)}*\;S_{\left(x,y,t\right)}$$

Where P*m* is the risk of infestation after the application of management activity *m*, and S_(x,y,t)_ is the probability that management activity m failed to eliminate the infestation, i.e., the probability of survey failure. In the case of surveys this would be captured by the probability that the survey failed to detect an infestation. In the case of full host removals, this would be the probability of failing to remove the beetle from the specified location.

Based on the assumptions that ground survey efficacy is 30%, aerial survey efficacy is 70%, and full host removal is 99.9% we can produce a series of conditional equations based on whether surveys and host removals are conducted at location (x,y) in year t such that, if a ground survey is conducted at (x,y,t) then:


Equation 4 $$Pg_{\left(x,y,t\right)}=\;P(x,y,t)*\;Sg_{\left(x,y,t\right)}$$

Where P*g*_(x,y,t)_ is the estimated probability of infestation at location (x,y) in year t after the completion of ground surveys, and S*g*_(x,y,t)_ is the probability of failure for a ground survey. However, the estimates of ground survey efficacy are based on a worst-case scenario, i.e., that surveyors are searching for a single oviposition site or exit hole on a single tree, and the limitations imposed by the portion of the tree that is visible from the ground. As beetle populations grow, so too would the amount of damage on the tree making the detection of the infestation on a given tree more likely as the size of the infestation within the tree increases. In cases where multiple surveys are conducted at a given location, the efficacy of the second survey may depend on the time elapsed since the location was previously surveyed. As a simple example, consider a single tree with a single exit hole, surveyed (but not detected) in year 1. If the same tree is surveyed again in year 2, the detectability of the infestation in the tree may be little changed. However, if 6 years pass between surveys, the beetle population on the tree will have gone through approximately three generations leading to an increase in the number of exit holes and oviposition pits within the tree. With increased signs of damage, the infestation would likely be easier to detect, and the probability of survey failure would decrease. Currently, the quantitative relationship between the inter-survey period and the increase in detection efficacy is not known. What is known, however, is the approximate generation time for each population based on phenology models for the Asian longhorned beetle (19). Based on this data we would expect the quantity of damage to roughly double every two years. To remain conservative, however, rather than doubling survey efficacy every 2 years, we add 5% to the efficacy for each additional year between surveys. While this is likely an overly conservative estimate it provides the integration of time-between-survey effects to the estimated changes in risk, though additional analyses are needed to refine this parameter. Using this approach, within any given year the probability of ground-survey failure is reduced based on the inter-survey period such that:


Equation 5 $$Sg_{\left(x,y,t\right)}=\;S\;–\;\left(0.05\;*\;I_{\left(x,y,t\right)}\right)$$

Where S*g*_(x,y,t)_ is the probability of ground survey failure in year t, S is the base rate of survey failure (0.7), and I*_x,y,t_* is the number of years since the prior survey of location (x,y) as of t. Similar adjustments may also be appropriate for aerial surveys, though at this time repeated aerial surveys are not common and are not included in these analyses.

Combining equations 4, 5 yields the equation to estimate the probability of infestation at (x,y,t) after the completion of ground surveys:


Equation 6 $$Sg_{\left(x,y,t\right)}=\;P_{\left(x,y,t\right)}*\;\left(S\;–\;\left(0.05\;*\;I_{\left(x,y,t\right)}\right)\right)$$

If a ground survey was not conducted at (x,y,t) then then the post-ground-survey risk (P*g*) at (x,y,t) is simply:


Equation 6’ $$Pg_{\left(x,y,t\right)}=\;P_{\left(x,y,t\right)}\;$$

If an aerial survey is conducted at (x,y,t),


Equation 7 $$Pga_{\left(x,y,t\right)}=\;Pg_{d}*\;Sa$$

Where P*ga*_(x,y,t)_ is the cumulative probability of infestation at location (x,y) in year t after the completion of both ground and aerial survey, and S*a* is the probability of failure for an aerial survey. If an aerial survey was not conducted at (x,y,t) then the post-ground-and-aerial-survey risk (P*ga*_(x,y,t)_) is simply:


Equation 7’ $$Pga_{\left(x,y,t\right)}=\;Pg_{\left(x,y,t\right)}$$

If full host removals are conducted at (x,y,t),


Equation 8 $$Pgar_{\left(x,y,t\right)}=\;Pga_{\left(x,y,t\right)}*\;Sr$$

Where P*gar*_(x,y,t)_ is the cumulative probability of infestation at location (x,y) in year t after the completion of ground and aerial surveys, and full host removals, and Sr is the probability of a full host removal to fully eradicate beetles from the location. If a full host removal was not conducted at (x,y,t),


Equation 8’ $$Pgar_{\left(x,y,t\right)}=\;Pga_{\left(x,y,t\right)}$$

P*gar*_(x,y,t)_ therefore represents the cumulative probability of infestation (risk) for location (x,y) at the end of year t following the completion of ground surveys, aerial surveys, and full host removals in year t. In the following year, the process is repeated, with P*gar*_(x,y,t)_ from the end of one year used to provide the P*gar*_(x,y,t-1)_ used to calculate A_(x,y)_ in the following year using Equation 1.

**Figure 2:**
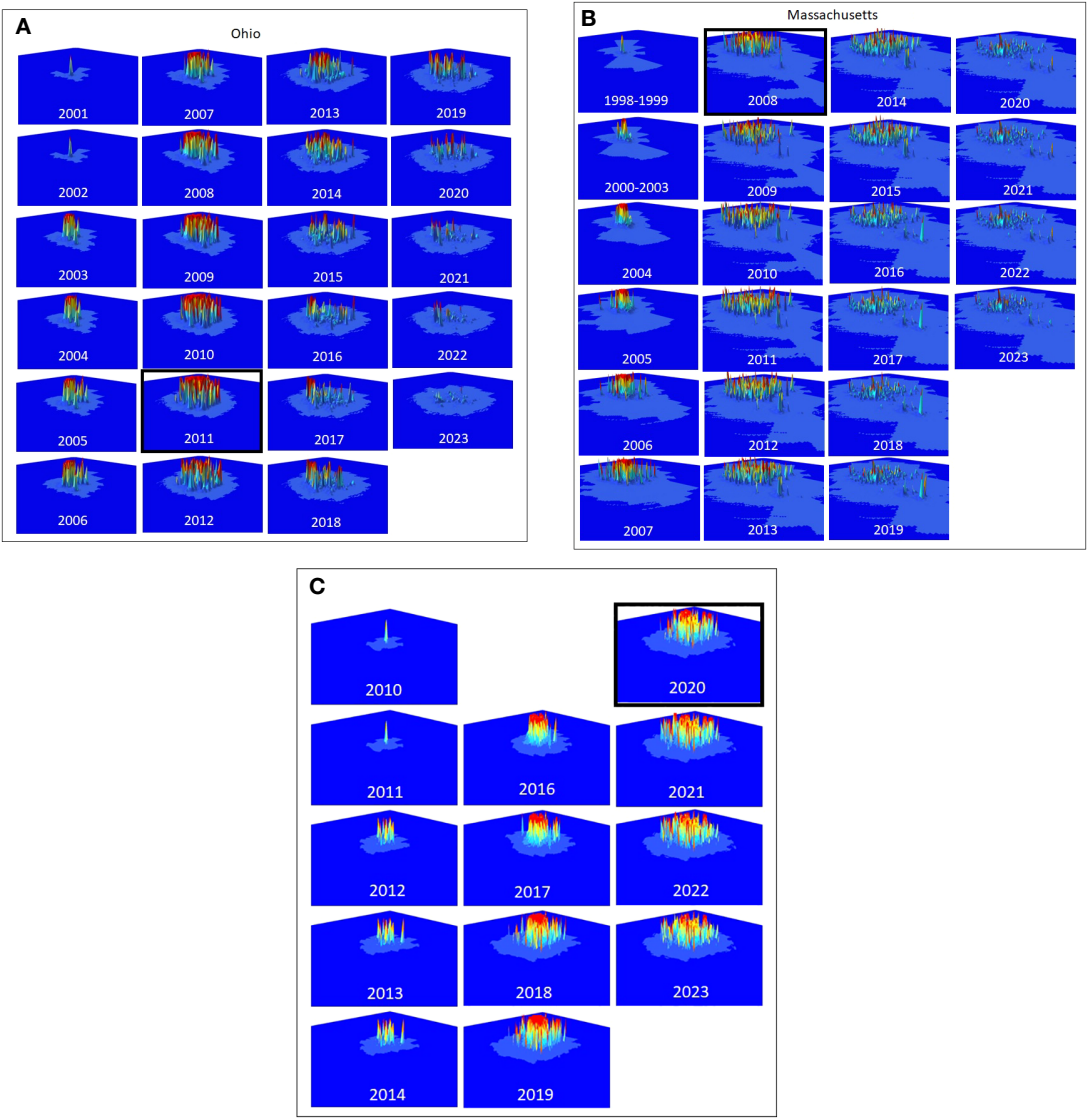
**(A–C)** The distribution and intensity of risk for Ohio, Massachusetts, and South Carolina are shown in the panels, starting with the estimated first year of the infestation. The year the infestation was detected is highlighted with a black box.

**Figure 3:**
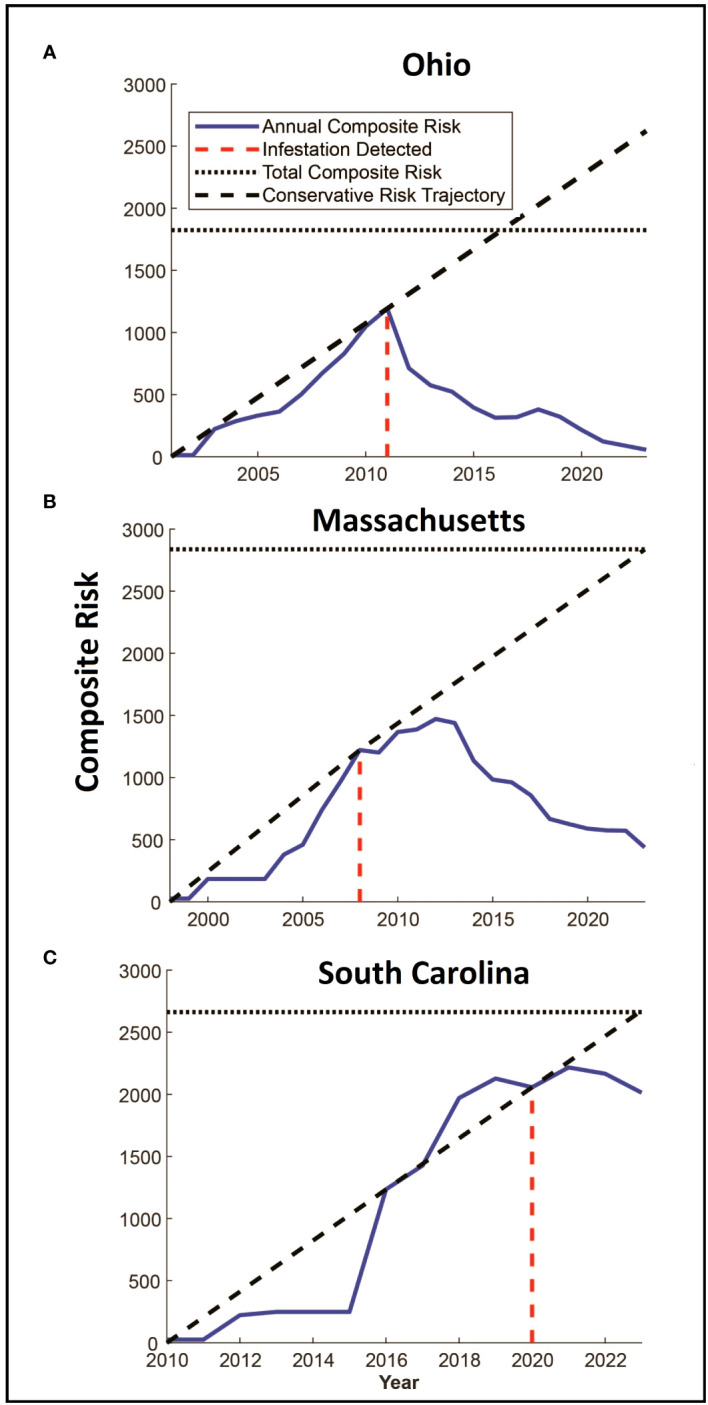
**(A–C)** Panels show estimated changes in composite risk through time for each of the three infested locations. The first year in each figure represents the estimated start date for the infestation, with the year of detection marked with the red dashed line. The solid blue line denotes the total composite risk for a location based on a combination of the accumulation of risk due to the infestation of trees, and the reduction in risk associated with eradication efforts. Note that risk accumulates up to the year of detection. The solid black dashed line represents a linear estimate of the accumulation of risk from the start of the infestation to the date of detection, extending this line beyond the date of detection provides a conservative indication of the potential accumulation of risk in a “no-management” condition. The horizontal dotted line represents the total quantity of accumulated risk that has been observed on the landscape and represents the risk that would be present if infested trees were removed, but surveys were not conducted.

Tracking Composite Risk Through Time – Quantifying the total quantity of risk on the landscape poses some challenges. A simple option is to calculate the probability that at least one hectare on the landscape is infested, which would be the product of the probabilities that each hectare is not infested (minus 1). This would provide an estimate of the probability that the landscape is infested however the result is limited to values between 0 and 1 and does not indicate the distribution or intensity of risk on the landscape. To capture the quantities, we use a risk index calculated as the sum of the probabilities of infestation for each hectare across the entire landscape. While the value is reduced to an index that does not represent a probability, it does provide a way to compare different quantities of overall of risk on the landscape such that large areas of low risk and small areas of high risk are not artificially weighted. This risk index can be represented graphically by plotting the hectares on a three-dimensional landscape in which the height of each raster is represented by the risk of infestation. The risk index or standing risk on the landscape would be the volume under the surface (such as those shown in **Figure 2**). By plotting this risk index through time (examples in **Figure 3**), we can quantify changes in the abundance and intensity of risk on the landscape as beetle populations spread via newly infested trees, surveys are conducted, and trees are removed.

## Results

**Figure 4:**
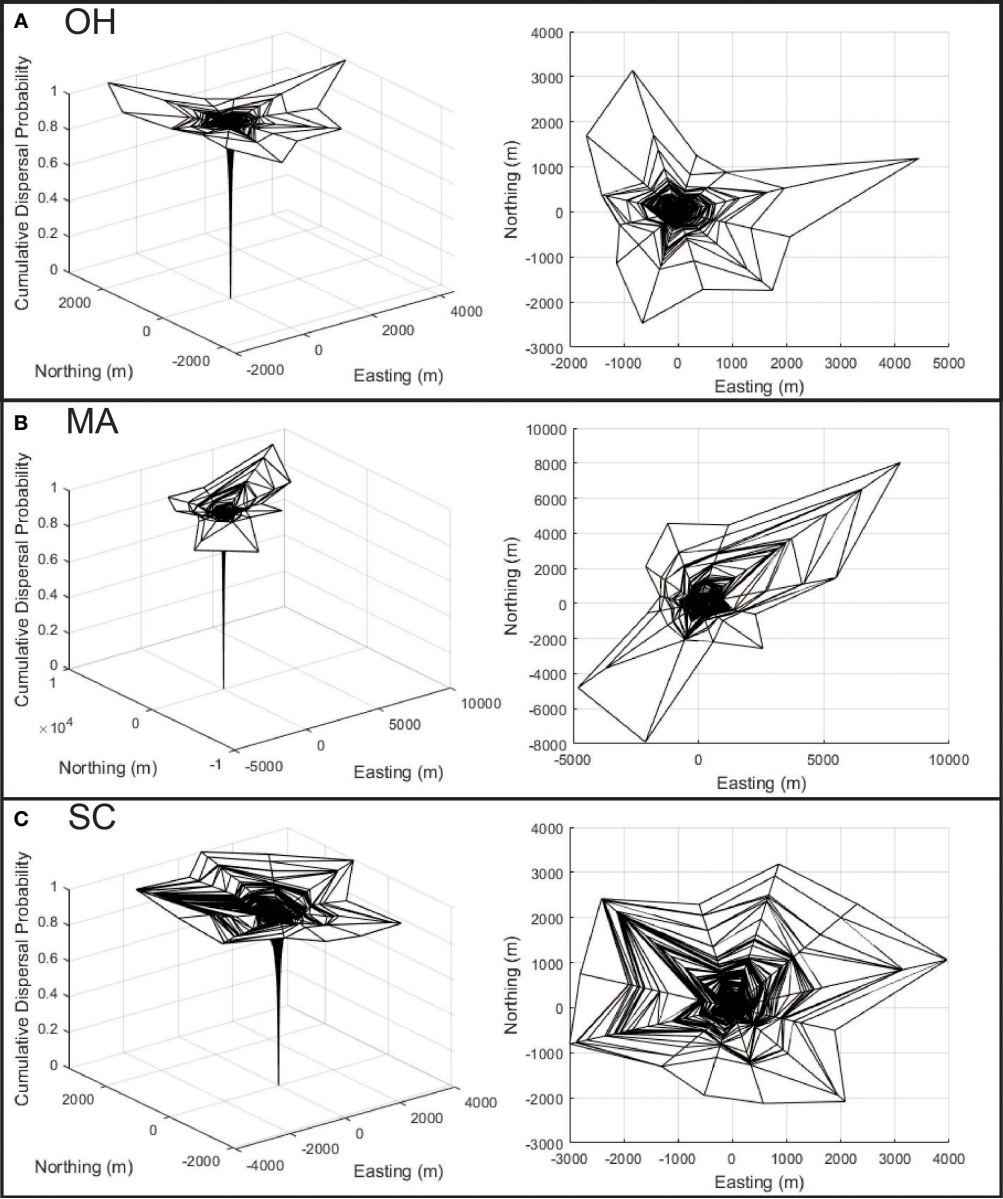
**(A–C)** Dispersal kernel surfaces are shown for each of the three analyzed infestations, with x and y axis representing east/west and north/south movement (respectively), with the cumulative probability of dispersal represented along the z axis. The panels along the left show the surface from an oblique perspective, while the panels on the right show the surface as viewed from the top to help illustrate anisotropy.

The reconstruction of Asian longhorned beetle dispersal kernels from Ohio (**Figure 4A**), Massachusetts (**Figure 4B**), and South Carolina (**Figure 4C**) show substantial variation among the locations with differences in both anisotropy and distance. The least isotropic population is found in Massachusetts (**Figure 4B**) where dispersal at long distances is strongly biased toward the northeast and southwest. The maximum inferred dispersal distance for this population is substantial at more than 7000 m, though it is important to note that the maximum distance is based on single longest dispersal event from a sample of 10,341 dispersal events. Dispersal along the northwest and southeast directions is shorter at approximately 2000 m. Dispersal by beetles in Ohio (**Figure 4A**) includes some anisotropy for the rare long-distance dispersing beetles with a bias towards the north and east, though much of the Ohio population (shown by the inner clines) demonstrates a more symmetrical pattern of dispersal. Maximum distances in Ohio exceed 3000 m, with some directions limited to just over 1000 m with dispersal patterns derived from 21,690 dispersal events. Dispersal in South Carolina (**Figure 4C**) is the most symmetrical of the three locations with maximum dispersal distances of roughly 3000 m in most directions based on 7,652 dispersal events. However, as the increased distance between the encircling clines in the dispersal kernel show, dispersal distances are more broadly distributed and include increased intermediate distances compared with the more skewed distribution of abundant short distance and rare long-distance movements exhibited by the other two populations. In each of the three infestations, the dispersal kernels follow the classic asymptotic distribution with most beetles dispersing within a relatively short distance with rare long-distance dispersal events.

**Figure 5:**
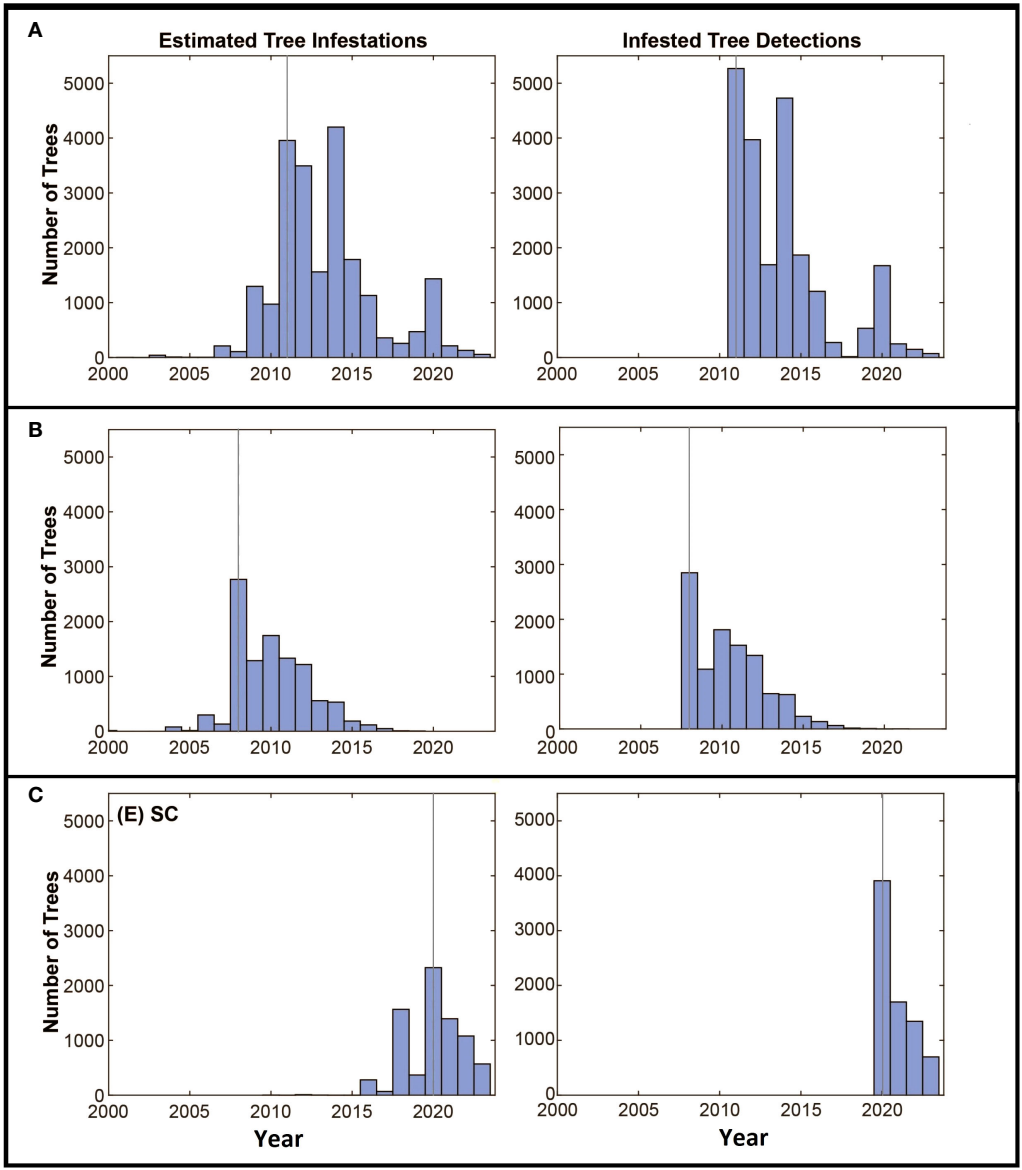
**(A–C)** Panels along the left show the estimated numbers of trees to become infested by year, the year the infestation was detected is marked with the grey vertical line. The detection of infested trees through time is shown in the right panel. Axes are standardized to allow comparisons among the locations.

The estimated dates of infestation for the trees in all three locations show some similarities through time (**Figures 5A–C**). In each case, populations start with a few infested trees with little increase in the number of infested trees over the first few years, followed by a rapid increase in the number of infested trees up to the date the infestation is detected. After detection and the initiation of eradication efforts, the number of newly infested trees declines over time. The rate of tree detections through time shows a similar pattern with most tree detections occurring early in the eradication program and the number of detections decreasing, presumptively as population growth and the infestation of new trees is reduced by the removal of infested source trees from the landscape.

The distribution and intensity of risk on the landscape are shown for Ohio, Massachusetts, and South Carolina (**Figures 2A–C**, respectively), and show both similarities and differences among the infestations. Each of the infestations shows a pattern of slow initial growth from the point of the initial infestation followed by a rapid increase in both the distribution and intensity of risk until the year in which the infestations were detected. Following detection, the intensity of risk is reduced in each of the infestations, though the portion of the landscape that includes at least some risk remains essentially unchanged. The three populations do differ however in the specific intensity and distribution of risk on the landscape, with the largest at-risk landscape found in Massachusetts (**Figure 2B**), and the largest area of high-intensity risk found in South Carolina **Figure 4C**) where eradication efforts have started most recently.

The total amount of risk (the sum of both risk intensity and distribution represented by the volume under the surfaces in **Figures 2A–C**) varies among the three infestations as shown in **Figures 3A–C**. In each of the three infestations the annual amount of standing risk on the landscape (represented by the blue line) has declined since the infestation was detected, though the decline in South Carolina (**Figure 3C**) is nominal, likely due to the relatively early state of the eradication program. The graphs include two additional lines that may be informative. The diagonal dashed line represents a very simplified extrapolation of the increase in risk on the landscape that might be expected under a “no-management” scenario and is based on the point of zero risk at the start of the infestation, and the presumed maximum risk observed at the time the infestation was first detected. The horizontal dotted line represents the total accumulation of risk that has ever existed on the landscape due to known infested trees but without the inclusion of risk reduction due to survey activities. The spaces between the lines represent risk prevented or removed. Using Ohio (**Figure 3A**) as an example, the space below the diagonal dashed line, and above the horizontal dotted line represents risk that was prevented from occurring through the removal of infested trees and the reduction of population growth and spread. The space below the horizontal dotted line and above the solid blue line represents risk that has been removed from the landscape via host removals and surveys. The space below the blue line represents remaining risk on the landscape. It is interesting to note that while the South Carolina eradication program is relatively young with fewer surveys completed on a smaller portion of the landscape relative to the other eradication programs, the cumulative risk index for the population exceeds a value of 2000. Ohio and Massachusetts with their larger landscapes hit maximum values of approximately 1200 and 1500, respectively. In the cases of both the Ohio and Massachusetts populations where eradication programs have been in place for 15 and 11 years, the quantity of standing risk on the landscape has been substantially reduced.

## Discussion

The analyses of the infestations in Ohio, Massachusetts, and South Carolina show that there is substantial variation in the patterns of dispersal among the infestations in both the distance and direction of beetle movements on the landscape. The distance and rate of dispersal play a significant role in both the spread (and functionally the control) of an invasive species. In their discussion of Allee effects and spread rates in invasive species, Liebhold and Tobin (13) noted the considerable variation in spread rates among species. The dispersal kernels described for these three locations also highlight the potential for significant within-species variation. The mechanisms that drive this variation are likely to include both physical landscape characteristics as well as climatic and potential anthropogenic factors and remain to be identified. However, even in the absence of knowledge of the mechanism driving the variation, it is quantifiable and can help optimize eradication efforts.

Prior studies on Asian longhorned beetles have used more direct methods to estimate dispersal kernels. Smith et al. (37, 38) for example conducted a large-scale mark-and-recapture study that included the release of 39,960 beetles in China in 2000. Of the release beetles, 395 were recaptured along transects yielding recaptures at distances of up to a kilometer. A dispersal kernel fitted to the recapture data indicated 98% of beetles would be expected to disperse less than 920 m, a distance somewhat shorter than many of the distances shown in **Figure 4**. However, it is important to note that there are substantial differences between the methods used by Smith et al. (37) and those described by Trotter III and Hull-Sanders (22) and used here. The data collected by Smith et al. (37) has the advantage of being based on direct experimental observation of dispersing beetles, while the methods used here are based on inference. Conversely the methods used here use the entire population of dispersing beetles (assuming one infested tree equals one dispersing beetle), while sample sizes in mark-and-recapture even in large studies such as Smith et al. (37, 38) are often limited, and rare long-distance dispersal events may have a disproportionately high probability of being missed. Despite these differences however, the order of magnitude of the dispersal distances observed in the two studies is quite similar suggesting general agreement between the studies, and these data may help fill the need for dispersal data in the U.S. where landscape heterogeneity may be higher, as discussed by Smith et al. (37).

The importance of quantifying local dispersal into eradication programs is highlighted by the variation in eradication strategies used in both Europe and the U.S. Current recommendations for survey boundaries in Europe are based on dispersal distances of 300 m (39) citing both the work by Smith et al. (37) and the work described by Favaro et al. (40). To our knowledge, the dispersal inference method described here has not been applied in European infestations, and so it is not possible to determine how the distances estimated for South Carolina, Ohio, and Massachusetts can be compared to those observed in Europe. In the U.S., the standard operating procedure for Cooperative Asian Longhorned Beetle Eradication Programs (33) call for surveys around infested trees in two areas identified as Level 1 and Level 2. Level 1 areas are those within a 804 meter (0.5 miles) buffer around all known infested trees, Level 2 includes the area within a 1,609 meter (1 mile) buffer around the outer perimeter of Level 1. Although these survey distances were selected prior to the availability of the analyses described here, their efficacy seems well supported by both these data and the successful eradication of multiple populations in the United States. These data also indicate it may be possible to refine survey distances to suit the dispersal observed in specific infestations in the U.S., creating opportunities to optimize individual programs.

The dispersal kernel surfaces in **Figure 4** show substantial variation in the directionality of dispersal among the locations, with implications for both the management of the species and for improving our understanding of the factors that structure dispersal. Using Massachusetts (**Figure 4B**) as an example, the data suggest that risk is likely to be extended along a northeast – southwest axis, and the application of surveys along this axis may accelerate the reduction in risk on the landscape (**Figure 2B**), and may increase the rate of infested tree detection. The presence of this bias may also suggest a mechanism, as in the area surrounding the Massachusetts infestation, prevailing winds during the summer tend to be along a northeast-southwest vector, suggesting winds may play a role in structuring long-distance dispersal events. In addition to facilitating surveys to reduce risk within the infestation, this information has the potential to guide monitoring activities outside the known infestation, though additional work is needed.

The estimate of the total risk on the landscape through time suggests there are some shared patterns among the eradication programs. Although the age of each program differs, each shows an increase in risk up to the date of detection and management, with management leading to reduced risk on the landscape as a whole as shown in **Figure 3**. In all three cases, the detection date coincides with a deflection point in the accumulation of risk, with risk being reduced as infested trees are removed, surveys are conducted, and the dispersal pressure on the landscape declines as shown by the decline in the number of newly infested trees shown in **Figure 5**. The decline is least evident in South Carolina however it is important to note that the eradication program in Carolina was initiated in 2020, and surveys have been conducted for only 3 years. As such, much of the landscape remains to be surveyed and more data will be needed to assess whether the apparent decline is sustained, though the patterns of decline associated with the initiation of management shown in the other two locations suggest the trend may continue.

The rates of risk reduction also vary among the infestations. The mechanisms that drive this variation merit further work, though the data shown here suggest factors that may play a role. Dispersal distance for example likely plays a significant role in both the distribution of risk and the evolution of risk on the landscape as eradication programs carry out management activities. For example, the risk index in Ohio appears to have declined rapidly since the detection of the infestation, relative to the risk in Massachusetts. Both infestations are of similar ages, however dispersal distances in Ohio have been shorter and the management area in Ohio is approximately half the size of the Massachusetts infestation. Additional work on the interplay between survey methods, landscape characteristics, and resources remains to be carried out, however the concentration of risk and reduction in dispersal distances may play a role in accelerating efforts to push populations of the Asian longhorned beetle to extinction.

A second point to note is that although the size of the infested landscape in South Carolina is similar to that of Ohio, and although more infested trees have been found in Massachusetts the composite risk in South Carolina is greater than either of the other two locations. The reasons for this are not known and will require the collection of additional information from South Carolina as surveys progress, but two factors seem plausible. First, the dispersal kernel for South Carolina describes maximum dispersal distances (shown by the outer bands of the dispersal kernels in **Figure 4**) similar to those observed in Ohio, however the distribution of dispersal events within these distances differ. The panels on the right side of **Figure 4** show the top-down view of the dispersal kernels for the three locations with lines that radiate from the center indicating the direction or dispersal, and the lines that make a circumference around the surface indicating the cumulative proportion of the population that disperses to that distance (with each line indicating one percentile of the population). In the case of the Ohio dispersal kernel, the lines are densely clustered at the center, with very few towards the edge suggesting few beetles travel at intermediate and long distances. The South Carolina figure however shows far more percentile clines at intermediate and longer distances, suggesting a larger portion of the population travels further than in Ohio, even if the maximum distances are similar. There are several mechanisms that may drive this, for example, the reduced density of maple on the landscape in South Carolina may force beetles to travel further to find suitable host trees. It may also be that the warmer temperatures in South Carolina are conducive to increased activity by the beetles, including longer flights. Work by Kappel et al. (3), conducted before the detection of the South Carolina population suggested populations in the south could have accelerated generation times, and it is possible that the reduced generation time leads to a shift in dispersal rate and distance, though more information is needed to link the characteristics of the landscape and the patterns of beetle dispersal.

The Asian longhorned beetle is a high-impact invasive that can be eradicated as shown by programs in Europe, Canada, and the United States. However, the process can take decades, which necessitates methods to track progress in eradication and to identify and evaluate the potential effect of future management activities on the infested landscape. The analyses described here based on ALBRisk 1.3 and ALBHMM 2.0 provide an opportunity for both, and the outputs shown in **Figure 3** may help assess eradication progress by quantifying both the current state of risk as shown in the blue line, but also the risk avoided and mitigated. The panels in **Figure 3** include two additional lines that describe these patterns. The horizontal dotted line represents the maximum quantity of risk that has accumulated on the landscape (i.e., risk that would be in place if no risk reduction by survey occurred), and a diagonal dashed line provides a rough estimate of the risk that might accumulate on the landscape if no management action were taken. The diagonal dashed line connects the point of zero risk at the start of the infestation, and the assumed maximum risk in the year the infestation was detected (before any management). The slope of the line connecting these points would be a very rough estimate of the rate of risk increase (though this is likely conservative as the relationship is shown as linear and risk likely increases exponentially). In combination, these lines represent rough projections of avoided or mitigated risk. The space below the diagonal line, and above the horizontal dotted line, for example, represents the increase in risk that has been avoided by carrying out eradication activities, and the space below the horizontal dotted line and the blue annual composite risk line represents the risk that has been actively removed from the landscape by surveys and host removals. Although coarse estimates, the figures overall suggest that the beetle population is being actively pushed away from establishment and towards local extinction, though additional work remains.

Future Utilities and Challenges– In addition to assessing the past and current progress in pushing an invasive species toward extinction, these analyses offer an opportunity to assess and optimize planned management efforts. The method described here uses records of survey activities and tree infestations to quantify changes in risk at a landscape-scale. By loading “records” of proposed management activities, the tool may have utility in estimating changes in risk based on those projected actions. The use of multiple proposed management plans could in turn be used to identify strategies that yield the greatest reduction in risk and may allow managers to “game” the landscape to optimize risk reduction and/or assess resources needed to achieve a given risk reduction. While work on this option remains to be done (including evaluations of how changes in the rate of future detections might alter projected risk reductions), the analyses provide a construct that can support efficient eradication.

While these tools provide new ways to assess and optimize surveys, key questions remain to be addressed. As noted previously there is substantial variation in the density, distribution, abundance, and composition of host trees both across and within each of the infested regions. Additional work is needed to continue to evaluate how each of these factors interacts with both the efficacy of surveys, and the probability of infestation within a given area, and it is hoped that ongoing work will continue to identify these interactions to both inform eradication programs and to expand the understanding of how landscapes and invasive species interact to structure invasions.

## Data availability statement

The data analyzed in this study is subject to the following licenses/restrictions: Data used was collected by federal eradication program managed by the Animal and Plant Health Inspection Service. Some data sets may include personally identifiable information. Additional information on gaining access to the data is available by contacting Josie.K.Ryan@usda.gov. Requests to access these datasets should be directed to Josie.K.Ryan@usda.gov.

## Author contributions

RT: Conceptualization, Formal Analysis, Investigation, Methodology, Project administration, Software, Supervision, Validation, Visualization, Writing – original draft, Writing – review & editing. JR: Data curation, Investigation, Resources, Writing – review & editing. JC: Conceptualization, Formal Analysis, Writing – review & editing. SP: Conceptualization, Data curation, Investigation, Methodology, Resources, Writing – review & editing.
